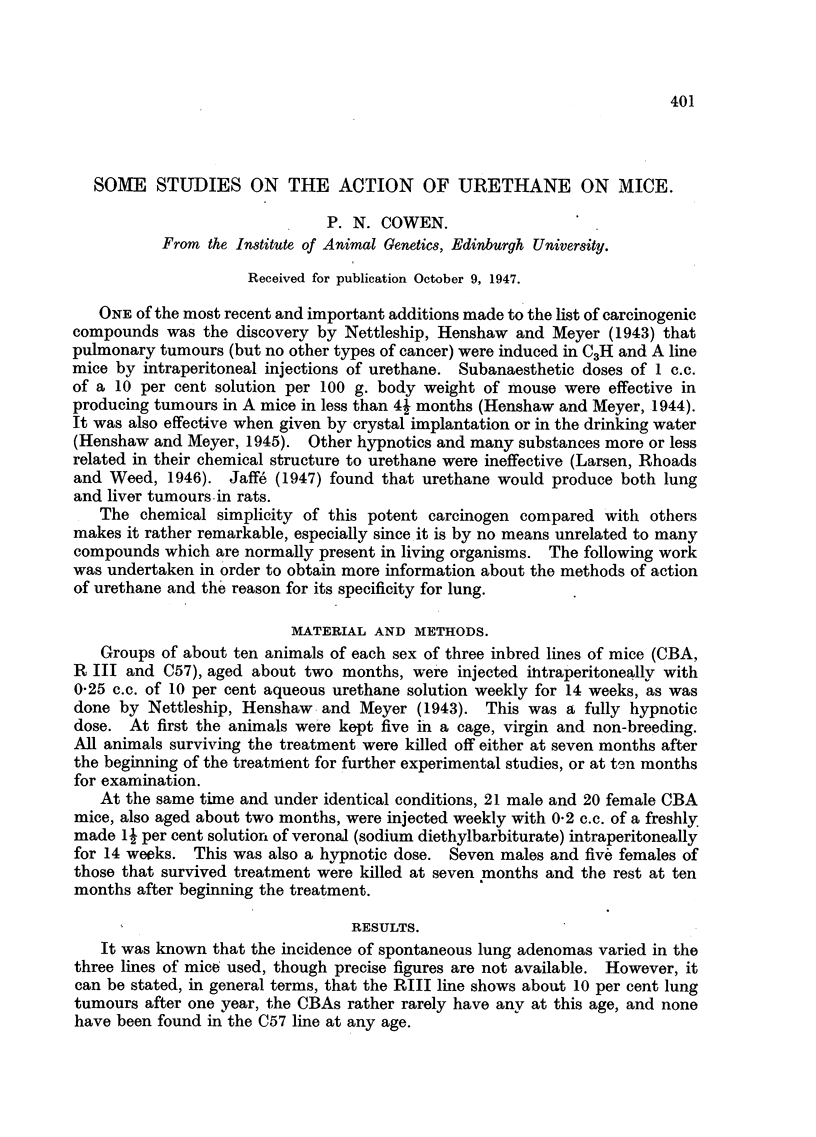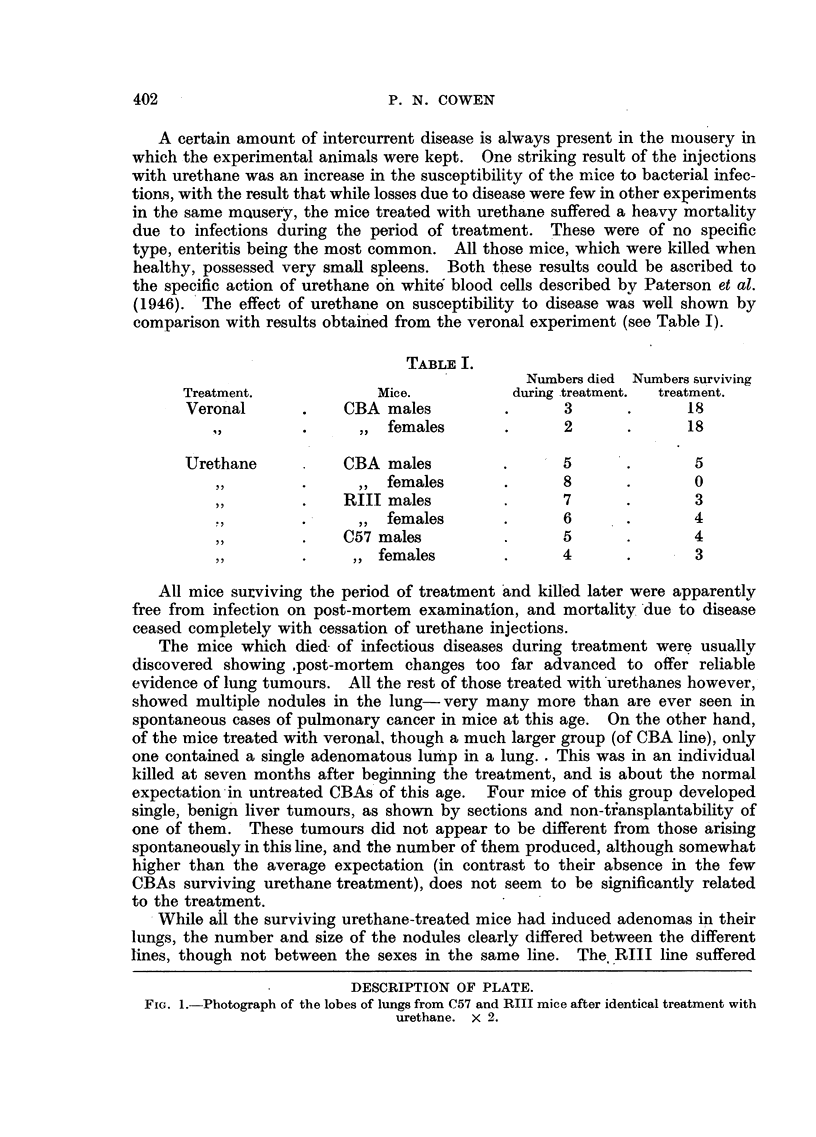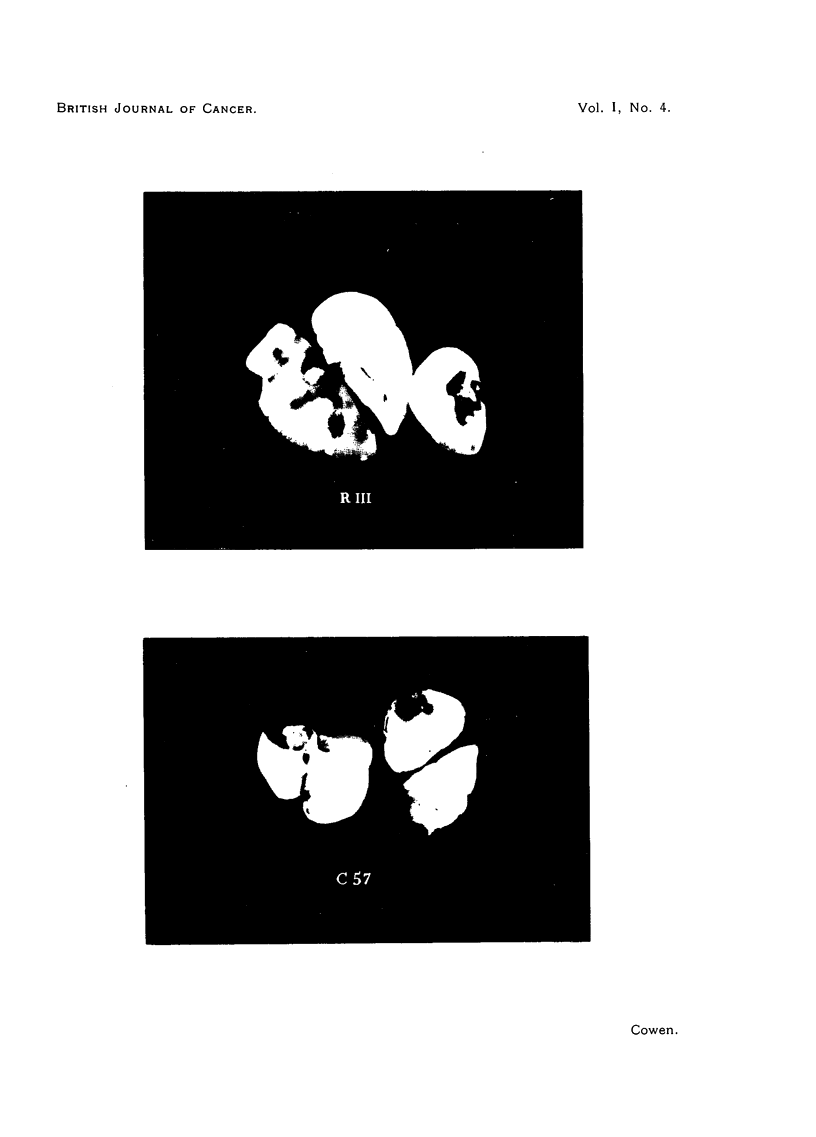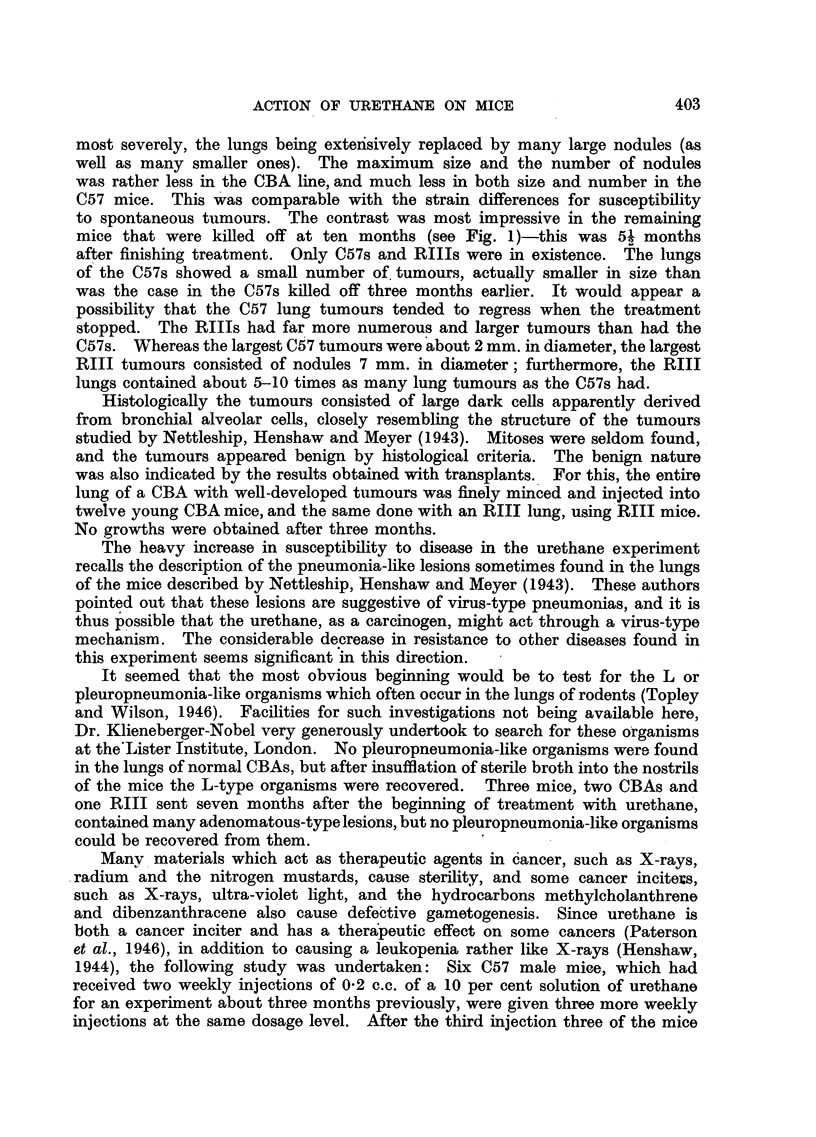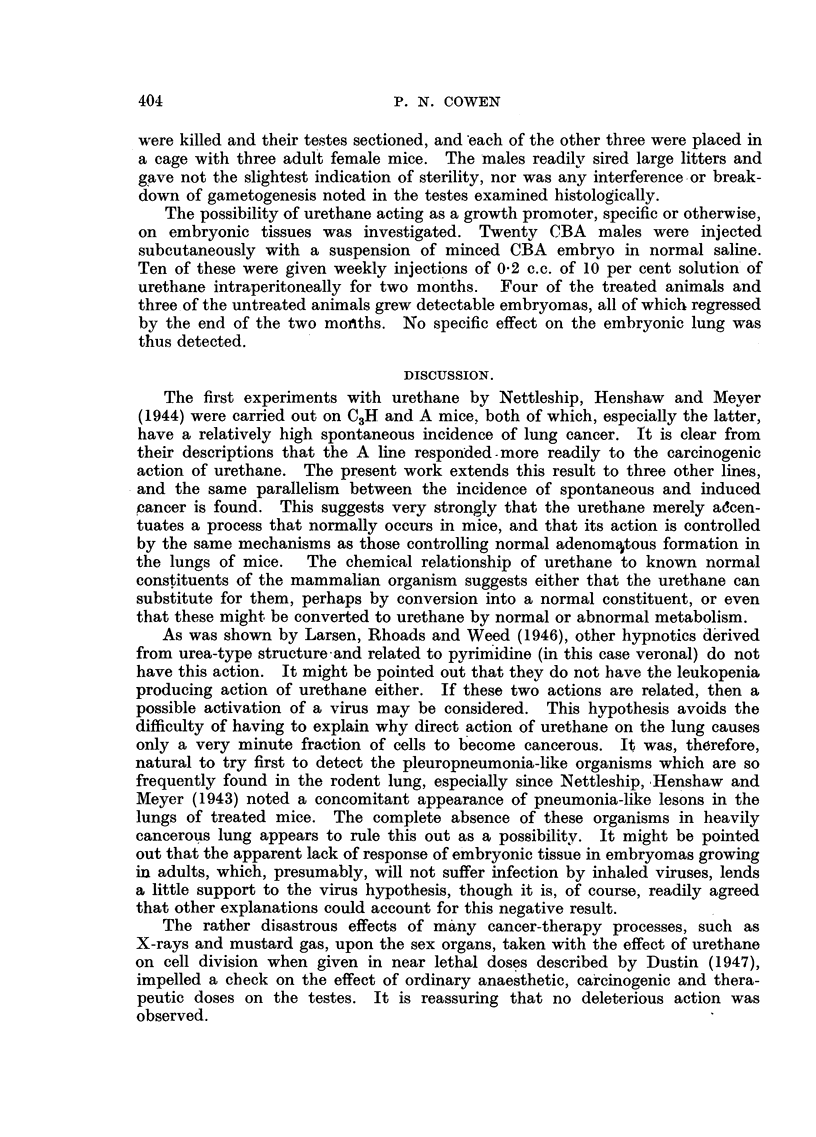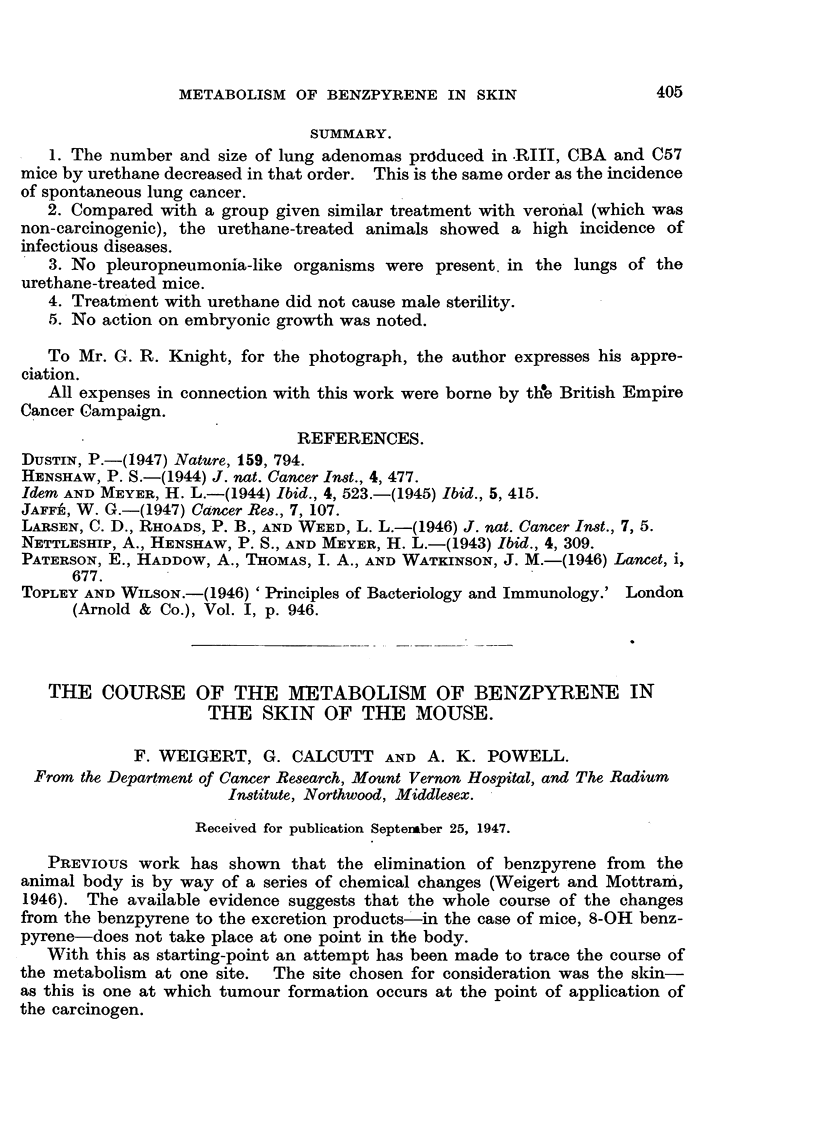# Some Studies on the Action of Urethane on Mice

**DOI:** 10.1038/bjc.1947.39

**Published:** 1947-12

**Authors:** P. N. Cowen

## Abstract

**Images:**


### SOME STUDIES ON THE ACTION OF URETHANE ON MICE.

P. N. COWEN.

*From the Institute of Animal Genetics, Edinburgh University.*

Received for publication October 9, 1947.

ONE of the most recent and important additions made to the list of carcinogenic compounds was the discovery by Nettleship, Henshaw and Meyer (1943) that pulmonary tumours (but no other types of cancer) were induced in $C_3H$ and A line mice by intraperitoneal injections of urethane. Subanaesthetic doses of 1 c.c. of a 10 per cent solution per 100 g. body weight of mouse were effective in producing tumours in A mice in less than 4½ months (Henshaw and Meyer, 1944). It was also effective when given by crystal implantation or in the drinking water (Henshaw and Meyer, 1945). Other hypnotics and many substances more or less related in their chemical structure to urethane were ineffective (Larsen, Rhoads and Weed, 1946). Jaffé (1947) found that urethane would produce both lung and liver tumours in rats.

The chemical simplicity of this potent carcinogen compared with others makes it rather remarkable, especially since it is by no means unrelated to many compounds which are normally present in living organisms. The following work was undertaken in order to obtain more information about the methods of action of urethane and the reason for its specificity for lung.

### MATERIAL AND METHODS.

Groups of about ten animals of each sex of three inbred lines of mice (CBA, R III and C57), aged about two months, were injected intraperitoneally with 0·25 c.c. of 10 per cent aqueous urethane solution weekly for 14 weeks, as was done by Nettleship, Henshaw and Meyer (1943). This was a fully hypnotic dose. At first the animals were kept five in a cage, virgin and non-breeding. All animals surviving the treatment were killed off either at seven months after the beginning of the treatment for further experimental studies, or at ten months for examination.

At the same time and under identical conditions, 21 male and 20 female CBA mice, also aged about two months, were injected weekly with 0·2 c.c. of a freshly made 1½ per cent solution of veronal (sodium diethylbarbiturate) intraperitoneally for 14 weeks. This was also a hypnotic dose. Seven males and five females of those that survived treatment were killed at seven months and the rest at ten months after beginning the treatment.

### RESULTS.

It was known that the incidence of spontaneous lung adenomas varied in the three lines of mice used, though precise figures are not available. However, it can be stated, in general terms, that the RIII line shows about 10 per cent lung tumours after one year, the CBAs rather rarely have any at this age, and none have been found in the C57 line at any age.

A certain amount of intercurrent disease is always present in the mousery in which the experimental animals were kept. One striking result of the injections with urethane was an increase in the susceptibility of the mice to bacterial infections, with the result that while losses due to disease were few in other experiments in the same mousery, the mice treated with urethane suffered a heavy mortality due to infections during the period of treatment. These were of no specific type, enteritis being the most common. All those mice, which were killed when healthy, possessed very small spleens. Both these results could be ascribed to the specific action of urethane on white blood cells described by Paterson *et al.* (1946). The effect of urethane on susceptibility to disease was well shown by comparison with results obtained from the veronal experiment (see Table I).

TABLE I.

| Treatment. | Mice. | Numbers died during treatment. | Numbers surviving treatment. |
|---|---|---|---|
| Veronal | CBA males | 3 | 18 |
| ,, | ,, females | 2 | 18 |
| Urethane | CBA males | 5 | 5 |
| ,, | ,, females | 8 | 0 |
| ,, | RIII males | 7 | 3 |
| ,, | ,, females | 6 | 4 |
| ,, | C57 males | 5 | 4 |
| ,, | ,, females | 4 | 3 |

All mice surviving the period of treatment and killed later were apparently free from infection on post-mortem examination, and mortality due to disease ceased completely with cessation of urethane injections.

The mice which died of infectious diseases during treatment were usually discovered showing .post-mortem changes too far advanced to offer reliable evidence of lung tumours. All the rest of those treated with urethanes however, showed multiple nodules in the lung—very many more than are ever seen in spontaneous cases of pulmonary cancer in mice at this age. On the other hand, of the mice treated with veronal, though a much larger group (of CBA line), only one contained a single adenomatous lump in a lung. . This was in an individual killed at seven months after beginning the treatment, and is about the normal expectation in untreated CBAs of this age. Four mice of this group developed single, benign liver tumours, as shown by sections and non-transplantability of one of them. These tumours did not appear to be different from those arising spontaneously in this line, and the number of them produced, although somewhat higher than the average expectation (in contrast to their absence in the few CBAs surviving urethane treatment), does not seem to be significantly related to the treatment.

While all the surviving urethane-treated mice had induced adenomas in their lungs, the number and size of the nodules clearly differed between the different lines, though not between the sexes in the same line. The RIII line suffered

DESCRIPTION OF PLATE.

FIG. 1.—Photograph of the lobes of lungs from C57 and RIII mice after identical treatment with urethane. × 2.

Cowen.

most severely, the lungs being extensively replaced by many large nodules (as well as many smaller ones). The maximum size and the number of nodules was rather less in the CBA line, and much less in both size and number in the C57 mice. This was comparable with the strain differences for susceptibility to spontaneous tumours. The contrast was most impressive in the remaining mice that were killed off at ten months (see Fig. 1)—this was 5½ months after finishing treatment. Only C57s and RIIIs were in existence. The lungs of the C57s showed a small number of tumours, actually smaller in size than was the case in the C57s killed off three months earlier. It would appear a possibility that the C57 lung tumours tended to regress when the treatment stopped. The RIIIs had far more numerous and larger tumours than had the C57s. Whereas the largest C57 tumours were about 2 mm. in diameter, the largest RIII tumours consisted of nodules 7 mm. in diameter; furthermore, the RIII lungs contained about 5–10 times as many lung tumours as the C57s had.

Histologically the tumours consisted of large dark cells apparently derived from bronchial alveolar cells, closely resembling the structure of the tumours studied by Nettleship, Henshaw and Meyer (1943). Mitoses were seldom found, and the tumours appeared benign by histological criteria. The benign nature was also indicated by the results obtained with transplants. For this, the entire lung of a CBA with well-developed tumours was finely minced and injected into twelve young CBA mice, and the same done with an RIII lung, using RIII mice. No growths were obtained after three months.

The heavy increase in susceptibility to disease in the urethane experiment recalls the description of the pneumonia-like lesions sometimes found in the lungs of the mice described by Nettleship, Henshaw and Meyer (1943). These authors pointed out that these lesions are suggestive of virus-type pneumonias, and it is thus possible that the urethane, as a carcinogen, might act through a virus-type mechanism. The considerable decrease in resistance to other diseases found in this experiment seems significant in this direction.

It seemed that the most obvious beginning would be to test for the L or pleuropneumonia-like organisms which often occur in the lungs of rodents (Topley and Wilson, 1946). Facilities for such investigations not being available here, Dr. Klieneberger-Nobel very generously undertook to search for these organisms at the Lister Institute, London. No pleuropneumonia-like organisms were found in the lungs of normal CBAs, but after insufflation of sterile broth into the nostrils of the mice the L-type organisms were recovered. Three mice, two CBAs and one RIII sent seven months after the beginning of treatment with urethane, contained many adenomatous-type lesions, but no pleuropneumonia-like organisms could be recovered from them.

Many materials which act as therapeutic agents in cancer, such as X-rays, radium and the nitrogen mustards, cause sterility, and some cancer inciters, such as X-rays, ultra-violet light, and the hydrocarbons methylcholanthrene and dibenzanthracene also cause defective gametogenesis. Since urethane is both a cancer inciter and has a therapeutic effect on some cancers (Paterson *et al.*, 1946), in addition to causing a leukopenia rather like X-rays (Henshaw, 1944), the following study was undertaken: Six C57 male mice, which had received two weekly injections of 0·2 c.c. of a 10 per cent solution of urethane for an experiment about three months previously, were given three more weekly injections at the same dosage level. After the third injection three of the mice were killed and their testes sectioned, and each of the other three were placed in a cage with three adult female mice. The males readily sired large litters and gave not the slightest indication of sterility, nor was any interference or breakdown of gametogenesis noted in the testes examined histologically.

The possibility of urethane acting as a growth promoter, specific or otherwise, on embryonic tissues was investigated. Twenty CBA males were injected subcutaneously with a suspension of minced CBA embryo in normal saline. Ten of these were given weekly injections of 0·2 c.c. of 10 per cent solution of urethane intraperitoneally for two months. Four of the treated animals and three of the untreated animals grew detectable embryomas, all of which regressed by the end of the two months. No specific effect on the embryonic lung was thus detected.

### DISCUSSION.

The first experiments with urethane by Nettleship, Henshaw and Meyer (1944) were carried out on $C_3H$ and A mice, both of which, especially the latter, have a relatively high spontaneous incidence of lung cancer. It is clear from their descriptions that the A line responded more readily to the carcinogenic action of urethane. The present work extends this result to three other lines, and the same parallelism between the incidence of spontaneous and induced cancer is found. This suggests very strongly that the urethane merely accentuates a process that normally occurs in mice, and that its action is controlled by the same mechanisms as those controlling normal adenomatous formation in the lungs of mice. The chemical relationship of urethane to known normal constituents of the mammalian organism suggests either that the urethane can substitute for them, perhaps by conversion into a normal constituent, or even that these might be converted to urethane by normal or abnormal metabolism.

As was shown by Larsen, Rhoads and Weed (1946), other hypnotics derived from urea-type structure and related to pyrimidine (in this case veronal) do not have this action. It might be pointed out that they do not have the leukopenia producing action of urethane either. If these two actions are related, then a possible activation of a virus may be considered. This hypothesis avoids the difficulty of having to explain why direct action of urethane on the lung causes only a very minute fraction of cells to become cancerous. It was, therefore, natural to try first to detect the pleuropneumonia-like organisms which are so frequently found in the rodent lung, especially since Nettleship, Henshaw and Meyer (1943) noted a concomitant appearance of pneumonia-like lesons in the lungs of treated mice. The complete absence of these organisms in heavily cancerous lung appears to rule this out as a possibility. It might be pointed out that the apparent lack of response of embryonic tissue in embryomas growing in adults, which, presumably, will not suffer infection by inhaled viruses, lends a little support to the virus hypothesis, though it is, of course, readily agreed that other explanations could account for this negative result.

The rather disastrous effects of many cancer-therapy processes, such as X-rays and mustard gas, upon the sex organs, taken with the effect of urethane on cell division when given in near lethal doses described by Dustin (1947), impelled a check on the effect of ordinary anaesthetic, carcinogenic and therapeutic doses on the testes. It is reassuring that no deleterious action was observed.

SUMMARY.

1. The number and size of lung adenomas produced in RIII, CBA and C57 mice by urethane decreased in that order. This is the same order as the incidence of spontaneous lung cancer.
2. Compared with a group given similar treatment with veronal (which was non-carcinogenic), the urethane-treated animals showed a high incidence of infectious diseases.
3. No pleuropneumonia-like organisms were present in the lungs of the urethane-treated mice.
4. Treatment with urethane did not cause male sterility.
5. No action on embryonic growth was noted.

To Mr. G. R. Knight, for the photograph, the author expresses his appreciation.

All expenses in connection with this work were borne by the British Empire Cancer Campaign.

REFERENCES.

DUSTIN, P.—(1947) *Nature*, **159**, 794.
HENSHAW, P. S.—(1944) *J. nat. Cancer Inst.*, **4**, 477.
*Idem* AND MEYER, H. L.—(1944) *Ibid.*, **4**, 523.—(1945) *Ibid.*, **5**, 415.
JAFFÉ, W. G.—(1947) *Cancer Res.*, **7**, 107.
LARSEN, C. D., RHOADS, P. B., AND WEED, L. L.—(1946) *J. nat. Cancer Inst.*, **7**, 5.
NETTLESHIP, A., HENSHAW, P. S., AND MEYER, H. L.—(1943) *Ibid.*, **4**, 309.
PATERSON, E., HADDOW, A., THOMAS, I. A., AND WATKINSON, J. M.—(1946) *Lancet*, i, 677.
TOPLEY AND WILSON.—(1946) 'Principles of Bacteriology and Immunology.' London (Arnold & Co.), Vol. I, p. 946.

---